# A Growth Modulation Index-Based GEISTRA Score as a New Prognostic Tool for Trabectedin Efficacy in Patients with Advanced Soft Tissue Sarcomas: A Spanish Group for Sarcoma Research (GEIS) Retrospective Study

**DOI:** 10.3390/cancers13040792

**Published:** 2021-02-14

**Authors:** Javier Martínez-Trufero, Luis Miguel De Sande-González, Pablo Luna, Javier Martin-Broto, Rosa Álvarez, Gloria Marquina, Roberto Diaz-Beveridge, Andrés Poveda, Juana María Cano, Josefina Cruz-Jurado, Antonio López Pousa, María Angeles Vaz Salgado, Claudia M. Valverde-Morales, Isabel Sevilla, Jerónimo Martínez-García, Jordi Rubio-Casadevall, Ana De Juan, Juan Antonio Carrasco, David S Moura, Ibon Gurruchaga-Sotes, Antonio Gutiérrez

**Affiliations:** 1 Javier Martinez-Trufero, Medical Oncology Department, Hospital Universitario Miguel Servet, 50009 Zaragoza, Spain; igurruchaga@salud.aragon.es; 2 Medical Oncology Department, Complejo Asistencial Universitario de Leon, 24008 Leon, Spain; lmgdesande@hotmail.com; 3 Medical Oncology Department, Hospital Universitario Son Espases, 07010 Palma de Mallorca, Spain; pablo.luna@ssib.es; 4 Medical Oncology Department, Hospital Universitario Virgen del Rocio, Instituto de Biomedicina de Sevilla (IBiS; CSIC, US, HUVR), 41013 Sevilla, Spain; jmartin@mustbesevilla.org; 5 Medical Oncology Department, Hospital Universitario Gregorio Marañon, 28009 Madrid, Spain; rosa.alvarez.al@gmail.com; 6 Medical Oncology Department, Hospital Universitario Clinico San Carlos, 28040 Madrid, Spain; gloriamarquina@gmail.com; 7 Medical Oncology Department, Hospital Politécnico La Fe, 46026 Valencia, Spain; robertdiazbeveridge@gmail.com; 8 Medical Oncology Department, Instituto Valenciano de Oncologia, 46007 Valencia, Spain; apovedav@gmail.com; 9 Medical Oncology Department, Hospital General de Ciudad Real, 13005 Ciudad Real, Spain; juanamariacano@gmail.com; 10 Medical Oncology Department, Hospital Universitario Canarias, 38320 Santa Cruz de Tenerife, Spain; jcruzjurado@gmail.com; 11 Medical Oncology Department, Hospital Universitario Santa Creu i Sant Pau, 08001 Barcelona, Spain; alopezp@santpau.cat; 12 Medical Oncology Department, Hospital Universitario Ramon y Cajal, RYCIS, CIBERONC, 28034 Madrid, Spain; mavaz4@gmail.com; 13 Medical Oncology Department, Hospital Universitario Vall D’Hebron, 08035 Barcelona, Spain; cvalverde@vhio.net; 14 Investigación Clínica y Traslacional en Cáncer, Instituto de Investigaciones Biomédicas de Málaga (IBIMA), Medical Oncology Department, Hospitales Universitarios Regional y Virgen de la Victoria de Málaga, 29010 Malaga, Spain; isevilla02@yahoo.es; 15 Medical Oncology Department, Hospital Virgen de la Arrixaca, 30120 Murcia, Spain; jeronimo@seom.org; 16 Medical Oncology Department, Instituto Catalan Oncologia, 17007 Girona, Spain; jrubio@iconcologia.net; 17 Medical Oncology Department, Hospital Marqués de Valdecilla, 39008 Santander, Spain; anade.juan@scsalud.es; 18 Medical Oncology Department, Hospital Alvaro Cunqueiro, 36213 Vigo, Spain; juan.antonio.carrasco.alvarez@sergas.es; 19 Instituto de Biomedicina de Sevilla (IBiS; CSIC, US, HUVR), 41013 Sevilla, Spain; david.moura@usal.es; 20 Hematology Department, Hospital Universitario Son Espases, 07010 Palma de Mallorca, Spain; antoniom.gutierrez@ssib.es

**Keywords:** trabectedin, sarcoma, growth modulation index, prognostic score, L-sarcoma, GEISTRA

## Abstract

**Simple Summary:**

Soft tissue sarcomas (STS) are an uncommon and heterogeneous group of tumors, with scarce options for treatment in advanced cases. There is no consensus regarding which is the best treatment sequence for these patients. Although trabectedin is an approved drug for STS treatment, after progression to anthracyclines, the clinical profile of the patients that most benefit from this drug it is not defined. We have retrospectively analyzed a sample of 357 nonselected sarcoma patients from real-world experience, treated homogeneously with trabectedin, confirming and validating results from previous clinical trials and other retrospective studies. After analyzing clinical prognostic factors, we selected those which predicted a better growth modulation index (GMI > 1.33), and we defined the GEISTRA score, an easy to obtain and reproducible clinical tool, that can help us to optimize the use of trabectedin in advanced sarcoma patients.

**Abstract:**

The aim of this study was to identify an easily reliable prognostic score that selects the subset of advanced soft tissue sarcoma (ASTS) patients with a higher benefit with trabectedin in terms of time to progression and overall survival. A retrospective series of 357 patients with ASTS treated with trabectedin as second- or further-line in 19 centers across Spain was analyzed. First, it was confirmed that patients with high growth modulation index (GMI > 1.33) were associated with the better clinical outcome. Univariate and multivariate analyses were performed to identify factors associated with a GMI > 1.33. Thus, GEISTRA score was based on metastasis free-interval (MFI ≤ 9.7 months), Karnofsky < 80%, Non L-sarcomas and better response in the previous systemic line. The median GMI was 0.82 (0–69), with 198 patients (55%) with a GMI < 1, 41 (11.5%) with a GMI 1–1.33 and 118 (33.1%) with a GMI > 1.33. The lowest GEISTRA score showed a median of time-to-progression (TTP) and overall survival (OS) of 5.7 and 19.5 months, respectively, whereas it was 1.8 and 3.1 months for TTP and OS, respectively, for the GEISTRA 4 score. This prognostic tool can contribute to better selecting candidates for trabectedin treatment in ASTS.

## 1. Introduction

Soft tissue sarcomas (STS) are an uncommon and heterogeneous group of 64 locally aggressive and/or malignant sarcoma subtypes according to the last WHO classification [1]. The number of STS histological subtypes has an increasing tendency, since new advances in pathology and molecular diagnosis have diversified and created new sarcoma entities [1]. In patients with unresectable and/or metastatic disease not amenable to curative surgery, the standard front-line treatment involves a palliative chemotherapy regimen with anthracyclines. Although there is some evidence that addition of ifosfamide to anthracyclines can achieve higher clinical benefits, no clear impact of such a combination was seen on overall survival (OS) [2,3] Beyond first-line treatment, several drugs and combinations have been widely introduced in daily clinical practice with different levels of activity and linked with specific sarcoma subtypes [4]. Nevertheless, there is no consensus regarding which is the best treatment sequence for patients with recurrent disease and different sarcoma histotypes to obtain optimal results.

Trabectedin (Yondelis^®^, PharmaMar, S.A., Madrid, Spain) I s a semisynthetic drug originally isolated from the Caribbean sea squirt *Ecteinascidia turbinata*. Trabectedin has a pleiotropic mechanism of action affecting key cell biology processes in tumor cells as well as in the tumor microenvironment with selective anti-inflammatory, immunomodulatory and antiangiogenic properties [5,6,7,8]. All these mechanisms contribute to a characteristic late response to trabectedin with a prolonged stabilization of tumor growth and dormancy of metastases. Trabectedin was the first marine-derived antineoplastic drug approved in 2007 in the European Union, and presently in about 80 countries across the world, for the treatment of patients with advanced STS (ASTS) who progressed after failure of anthracyclines and ifosfamide, or for those patients who are unsuitable to receive these agents [9]. In 2015, trabectedin was also approved in the U.S. for patients with advanced liposarcoma or leiomyosarcoma (commonly referred as L-sarcomas) based on the results of a pivotal, randomized, phase III study that evaluated the efficacy and safety of trabectedin as compared with dacarbazine, an active comparator used in the treatment of patients with ASTS [10].

Regarding the assessment of clinical benefit, the selection of clinically meaningful scientific objectives and standardized study endpoints for recurrent disease is critical. Nowadays, tumor growth delay seems to be a more informative objective than mere tumor shrinkage. Therefore, time to event outcomes such as time to progression (TTP) and progression-free survival (PFS) are considered as the preferred primary endpoints in sarcoma trials [11]. In 1998, Von Hoff described an approach based on the use of intrapatient comparison of successive TTP intervals where each couple tumor/patient acts as its own control [12]. He defined the growth modulation index (GMI) as the ratio of the TTP with a determined line of treatment (TTPn) divided by the TTP from the previous line of treatment (TTPn-1). Since the successive TTPs tend to be shorter in subsequent treatment lines, it has been suggested that GMI > 1.33 is the threshold that defines a drug as an agent of excellent efficacy [13,14]. Thus far, this approach was used to evaluate the efficacy of trabectedin in ASTS in two retrospective and one prospective study, confirming that GMI is a useful exploratory efficacy endpoint and a good surrogate marker of drug activity, which also considers the heterogeneity of each STS [13,14,15].

Based on the results of a preliminary analysis of data from 198 patients with ASTS treated with trabectedin (training cohort), we previously defined a new GEISTRA score, identifying L-sarcomas, metastatic-free interval (MFI) from initial diagnosis and Karnofsky performance status (KS) as independently associated prognostic variables associated with high GMI of >1.33 [16]. In this retrospective study, we have further analyzed data from 191 other patients considered as the validation cohort (total population: *n* = 357), with the aim of validating the GEISTRA score and additionally characterizing a clinical profile of the patients that may benefit most from trabectedin.

## 2. Materials and Methods

### 2.1. Database and Objectives

We carried out a retrospective study of the Spanish Group of Sarcoma Research (GEIS) registry database with real-life patients’ data treated with trabectedin between January 2007 and June 2016. This trial was implemented in 18 representative GEIS centers with the aim to have a good geographical representation of patients across Spain. The primary objective of the study was to identify which group of patients with ASTS benefits most from trabectedin given as a second- or later-line chemotherapy by evaluating the concordance among the GMI > 1.33, response and survival outcomes, and the clinical characteristics of patients. Secondary endpoint was to assess the efficacy of trabectedin according to histological sarcoma subtype.

All study procedures were conducted in accordance with the ethical standards as laid down in the 1964 Declaration of Helsinki and its later amendments, guidelines for Good Clinical Practice and were approved by the institutional review boards of each participating center. All reasonable efforts to obtain signed informed consent forms from all study participants to retrieve their data and tumor samples were done before study registration. All participant centers had to obtain the approval of ethics committees before registration.

### 2.2. Patients and Treatments

All eligible patients had to be on treatment with trabectedin and have received a minimum of one cycle of trabectedin as second- or later-line treatment before their inclusion in the study. Eligible patients were adults (>18 years old) with histologically proven and measurable ASTS who received an anthracycline-based treatment as first-line treatment, and with data available to calculate survival outcomes. Patients who had received an anthracycline as neo- and/or adjuvant treatment, and subsequently received trabectedin as first advanced chemotherapy for recurrent/metastatic disease, were not included in the analysis. Exclusion criteria included patients with contraindications to the use of trabectedin as defined in the marketing authorization, patients with gastrointestinal stromal tumor or bone sarcoma, and pregnant and breastfeeding women.

Trabectedin was administered in accordance with the marketing authorization at the recommended dose of 1.5 mg/m^2^ body surface area (BSA), administered as an intravenous infusion over 24 h with a 3-week interval between cycles. Pretreatment with corticosteroids (e.g., dexamethasone 20 mg intravenously 30 min before trabectedin) was usually prescribed for all patients receiving trabectedin.

### 2.3. GEISTRA Score Design

To develop the new score, the series was split into training and validation cohorts. The training set was the original one which the model was stemmed from l [16]. The optimal cutoff of the quantitative variable metastatic-free interval (MFI) was calculated through Receiver Operating Curves (ROC). All the remaining patients included in the registry after those included in the training set, were used as validation set.

The GMI was calculated as defined by Von Hoff [12] and was expressed as a ratio of intrapatient successive TTPs: GMI = TTP under trabectedin/TTP for treatment prior to trabectedin. TTPs were supplied by investigator centers, and there was no central review. The TTP for treatment prior to trabectedin was calculated from the start date of prior chemotherapy treatment to the date of progressive disease. To build a new GEISTRA score in the training cohort, first we analyzed which independently associated prognostic variables could predict a GMI > 1.33, indicating the highest clinical benefit from the treatment with trabectedin. Subsequently, we assigned one point for each adversely affected variable to produce the final rate of GEISTRA score, ranging from 0–4 points.

### 2.4. Statistical Methods

Variables following binomial distributions are expressed as frequencies and percentages, whereas categorical variables are expressed as absolute and relative frequencies or continuous variables as the median, range (minimum–maximum). Comparisons between qualitative variables were done using the Fisher Exact Test or Chi-square. Comparisons between quantitative and qualitative variables were performed through nonparametric tests (U of Mann–Whitney or Kruskal–Wallis tests). Multivariate analysis of the relationship between qualitative and binary variables was made with binary logistic regression to identify and characterize the subgroup of patients with GMI > 1.33 (i.e., possible prognostic factors).

The objective response rate (ORR) of trabectedin was evaluated according to Response Evaluation Criteria in Solid Tumors (RECIST) v.1.1 [17]. Moreover, the disease control rate (DCR) was defined as the percentage of patients with a complete response (CR) or partial response (PR) and/or stable disease (SD). Time-to-event endpoints and their fixed-time estimations were estimated according to the Kaplan–Meier method and were compared using the log-rank test. The TTP and OS analyses were defined as the time interval from the date of diagnosis, metastasis or first administration of trabectedin to the earliest date of disease progression or disease-related death as reported by the investigator for TTP, whereas OS was defined as the time between the start of trabectedin and patient death from any cause. Multivariate survival analysis with the variables that proved to be significant in univariate analysis was performed according to the Cox proportional hazard regression model. All *p*-values reported were two-sided, and the significance level selected was 0.05.

## 3. Results

### 3.1. Characteristics of Patients and Treatments

We collected data from 387 patients with ASTS enrolled by 19 GEIS centers across Spain. Of those, 30 patients were considered as noneligible for analysis as they underwent surgery between the prior chemotherapy and trabectedin and, thus, their GMIs could not be properly calculated. Therefore, data from 357 patients were included in the analysis set. Table 1 shows the whole series as well as training and validation cohorts. At diagnosis patients had a median age of 50 years (range: 14–79 years), slightly more than half were women (52.7%), and most had nonmetastatic disease (77.3%). L-sarcomas (54.1%) were the most prevalent histological types of sarcomas (leiomyosarcoma 31.7%; liposarcoma 22.4%).

All patients were pretreated with an anthracycline-based chemotherapy regimen, 133 (37.3%) as first-line treatment for metastatic STS, whereas the rest were treated in the adjuvant setting. Additionally, 115 patients (32.2%) were also pretreated with gemcitabine-based chemotherapy. Patients received a median of 4 trabectedin cycles per patient (range: 1–42). Overall, 154 patients (43.1%) received trabectedin as second-line chemotherapy, 15 of whom immediately after anthracycline-based treatment. The rest of the patients received trabectedin either as third- (*n* = 135, 37.8%) or fourth-line (*n* = 68, 19%) chemotherapy.

### 3.2. Response to Treatment and Survival Analysis

A total of 325 patients (91.0%) were evaluable for efficacy according to RECIST, given that 27 patients were treated with ≤2 trabectedin cycles and global deterioration of the health status requiring discontinuation of the treatment before any assessment in nine patients. Five patients (1.5%) had a complete response (CR) and 36 patients (11.1%) achieved a partial response (PR), reaching an ORR of 12.6%. CR cases were achieved in patients with the following histotypes: 3 liposarcomas (2 myxoid, 1 dedifferentiated), and 2 synovial sarcoma patients.

Additionally, 115 patients (35.4%) had stable disease as best response for a DCR of 48.0% (*n* = 156). All other evaluable patients (*n* = 169, 52.0%) showed progression as the best response.

Considering the whole series, after a median follow-up of 75.1 months (range: 8.4–286.1) from initial diagnosis, treatment with trabectedin resulted in a median OS of 12.0 months (95% CI: 10–13.9) in the whole population and 17.9 months (95% CI: 14.3–21.5) in patients with L-sarcomas. Median OS from initial diagnosis and metastatic disease was 42.9 months (95% CI: 37.7–48) and 27.7 months (95% CI: 24.5–30.9), respectively. Patients with L-sarcomas compared to patients with non-L-sarcomas obtained larger median OS from initial diagnosis (55.8 months (95% CI: 46.6–65.1) vs. 34.8 months (95% CI: 28.6–41)) and from metastatic disease diagnosis (33.9 months (95% CI: 29.1–38.6) vs. 21.4 months (95% CI: 18.2–24.6)).

The median TTP for the immediately prior chemotherapy line was 2.6 months (95% CI: 2.4–2.8), whereas median TTP for trabectedin was 3.5 months (95% CI: 2.8–4.0). Median TTP from trabectedin significantly differed (*p* < 0.001) in patients with L-sarcomas as compared with patients with non-L-sarcomas (5.1 months [95% CI: 3.8–6.4] vs. 2.8 months [95% CI: 2.3–3.3]). Exploratory univariate and multivariate analyses identified metastasis-free interval (MFI) <10 months, Karnofsky performance status <80%, non-L-sarcoma and grade 3 sarcoma as per the French Federation of Cancer Centers Sarcoma Group (FNCLCC) criteria as independent prognostic factors associated with both worse TTP and OS (Table 2).

### 3.3. GEISTRA Score

In the training cohort (*n* = 191), the median GMI (mGMI) was 0.91 (0–69). Overall, 101 patients (52.9%) had a GMI < 1, 22 patients (11.5%) a GMI equal to 1–1.33 and 68 (35.6%) had a GMI > 1.33. We found a statistically significant association between the GMI > 1.33 and median OS and TTP (*p* < 0.001; Table 3). There was also a high concordance rate between the best objective response to trabectedin and the GMI > 1.33 (*p* < 0.001; Table 3).

Table 4 depicts the results of the univariate logistic analysis of clinical prognostic factors related to GMI in the training cohort. Variables found to be significantly different in the univariate analysis were included in multivariate analysis. Four variables were found to be independently associated to GMI > 1.33: MFI > 5, Karnofsky performance status >80%, L-sarcoma histology, and progression after the prior chemotherapy line. Those factors were included in a multivariate analysis and also resulted to be significantly associated to GMI > 1.33 (Table 4).

**Figure 1:**
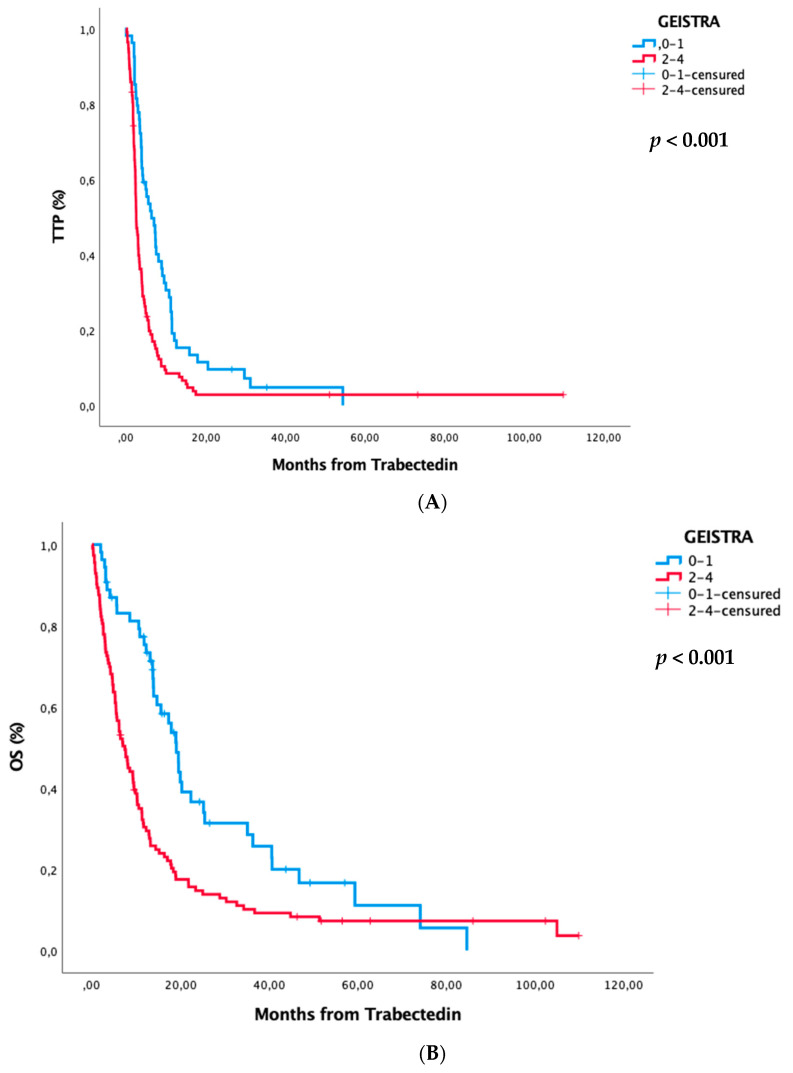

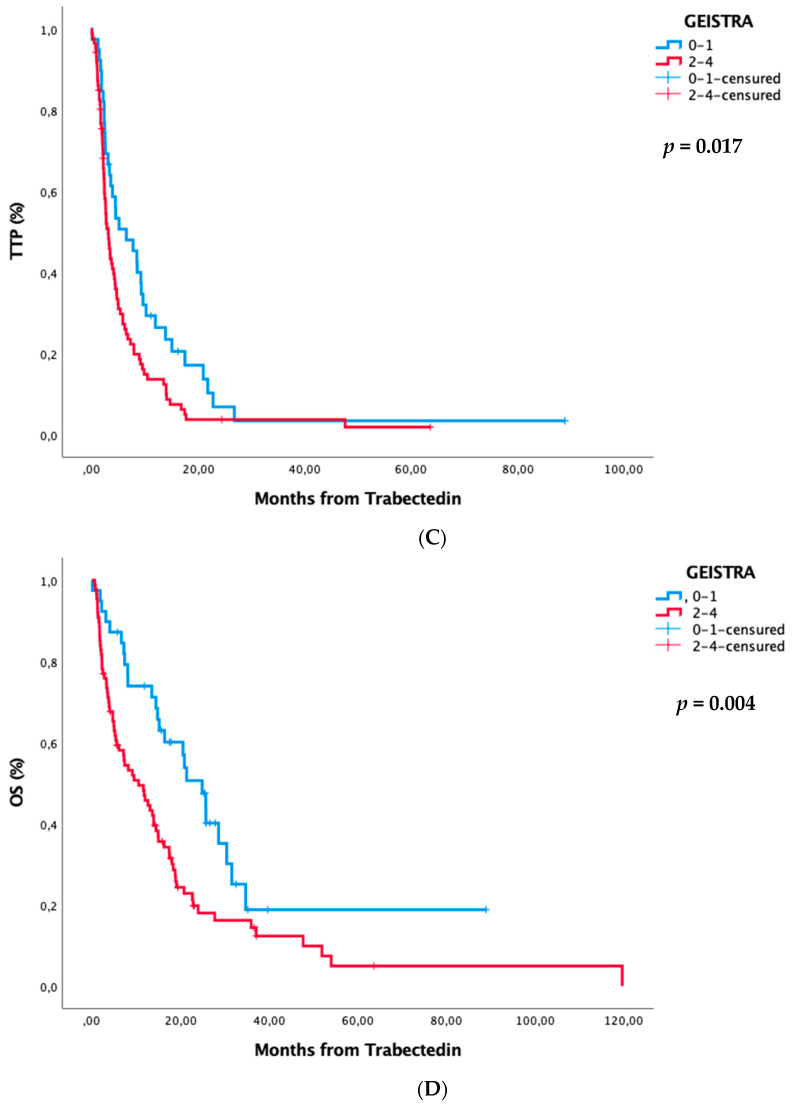
Time to progression and overall survival curves according to GEISTRA score in the training (**A**,**B**) and validation (**C**,**D**) cohorts.

The GEISTRA score was defined taking into account these four variables, assigning one point for each variable: MFI 0–5 months, Karnofsky performance status <80%, non-L-sarcoma histology, and clinical benefit (i.e., CR/PR/SD) of previous line. Based on those variables, we defined three prognostic staging groups: a GEISTRA group 0 for a total score between 0–1 points and group 1 for a score of 2–4 points. Finally, we observed a statistically significant correlation between the lower scores of the two-stage GEISTRA score and larger median TTP and median OS (*p* < 0.001) following the treatment with trabectedin as shown in Table 5 and Figure 1. We validated the score using the validation cohort as we can see in Figure 1.

## 4. Discussion

This retrospective analysis of clinicopathological prognostic variables aimed to identify patients obtaining a higher benefit with trabectedin treatment as second or further line for progressing ASTS. With this in mind, a new GMI-based score, GEISTRA, which showed a strong correlation with clinical efficacy endpoints (ORR, TTP and OS) was defined. We previously defined this score in a training cohort, and validated with an additional validation cohort [16]. Specifically, the independent worse prognostic variables comprising the GEISTRA score were MFI < 5 months, Karnofsky < 80%, Non L-sarcomas and obtaining clinical benefit rate in the previous systemic line.

MFI was identified as a prognostic factor for first and second lines in advanced STS by EORTC trials [18]. In fact, MFI was an independent prognostic variable for a better progression free survival in ASTS patients treated with a second or further line [19]. A longer MFI could reflect a more indolent tumor biological behavior that could be related with a slower proliferation also in advanced disease. In line with that, the median time to response for trabectedin ranges from 3.7 to 5.3 in prospective phase II trials [20,21], which could indicate that indolent tumors are a favorable profile for trabectedin efficacy.

Despite the fact that trabectedin can be effective across a wider range of sarcoma subtypes such as translocation-related sarcomas, undifferentiated pleomorphic sarcomas or synovial sarcoma [22,23], the fact is that pivotal trials of trabectedin have been conducted in L-sarcomas, precisely because a greater benefit of trabectedin is obtained in this context [10]. Myxoid liposarcoma is a particularly sensitive subtype to trabectedin, where a new mechanism of action was described for this drug through the displacement of oncogenic transcription factor from the target promoter [24]. Even though other histologic grouping could be performed, it was preferred to consider non-L vs. L-sarcomas as roughly half of patients were distributed in each group.

Not surprisingly, performance status at the time of trabectedin initiation resulted in an independent prognostic variable. Performance status has shown to be a robust prognostic variable in ASTS [25]. In reality, the fact that response probability and the overall survival are shortened with increasing systemic lines in ASTS [26] could be related to an impairment of performance status.

More remarkable is the variable related to obtaining any response or stabilization with the right previous line as a worse prognostic factor. This obviously has to do with some selection bias inherent to targeting the population with GMI > 1.33. Nevertheless, analyzing the median TTP in patients with progressive disease or the objective response to the previous line, they were almost similar (9.9 vs. 16 months), so we do not consider that its influence on GMI is so remarkable. On the other hand, what we consider clinically relevant, and noteworthy to take into consideration, is the fact that progressive disease, as the best RECIST response in the previous systemic line, does not preclude trabectedin efficacy.

Being the ORR below 10% for the registered drugs in second lines of ASTS, other prognostic tools, such as GEISTRA, showing a good correlation to PFS and OS appear appealing.

To the best of our knowledge, the combination of these clinical parameters, widely and easily available in clinical practice, has not been previously studied in a large cohort of treated ASTS patients.

In the present study trabectedin administration resulted in an ORR of 12.6%, DCR of 48.0%, and a median TTP and OS of 3.5 and 12.0 months, respectively. These figures are similar to previously reported clinical trials with trabectedin in ASTS [9,10,15,23] as well as to retrospective studies [27,28,29,30] (Supplementary Table S1). It is noteworthy that despite our series containing 45.9% of non-L-sarcomas, clinical endpoints were similar to series only focusing on L-sarcomas. Nevertheless, slightly better results than published have been found in our series among L-sarcomas for both, median TTP and OS. Generally, there are sparse data addressing prognostic or predictive scores in ASTS, as just a few studies have previously described prognostic scores and typically among patients with localized disease [31,32]. For instance, Penel et al. reported the statistically significant relationship between a high GMI and favorable efficacy outcomes in patients treated with trabectedin (i.e., ORR, TTP and OS) [14]. High GMI rates seen for trabectedin in the present study (GMI 1–1.33: 11.5%; GMI > 1.33: 33.1%) favorably compare to Penel study [14] which reported 7.5% and 29.0% of patients with a GMI of 1–1.33 and a GMI > 1.33, respectively. Besides, Cousin reported a significant correlation between those with GMI > 1.33 and OS in a retrospective multicenter study in patients with ASTS receiving an active second-line after doxorubicin-based regimens [13]. It is noteworthy that the median GMI (mGMI) of 0.82 obtained in this study is in the range of other series treated with trabectedin, such as those reported by Penel et al. (mGMI: 0.6) [14], Buonadonna et al. (mGMI:0.8) [15] Cousin et al. (mGMI:0.75) [13] and Kobayashi et al. (mGMI:0.91) [30], despite the large proportion of patients (45.9%) with non-L sarcomas. This indicates that a consistent benefit is reached with trabectedin in a substantial number of patients in second line of ASTS. Considering that median of PFS is decreasing as the number of lines in ASTS (and in general in all tumors) increases due to more aggressive tumor phenotype and to the more fragile host, a median GMI close to the unit indicates a very good option for a drug prescribed at least in second line of ASPS.

In contrast to previous findings regarding a lower trabectedin efficacy in the context of a higher number of previous lines [32], we did not find this factor of prognostic relevance. However, it should be considered that those patients receiving trabectedin as a first line of ASTS, as they had received anthracyclines and ifosfamide in a perioperative setting, have not been included in our study.

Gemcitabine-based treatment is one of the most widely used second-line chemotherapy schedules in ASTS. In our study, previous administration of gemcitabine-based treatment apparently did not have any influence on trabectedin efficacy, as we found no correlation between gemcitabine treatment and GMI, nor any significant prognostic value.

The fact of having selected variables related to patients with GMI > 1.33 can be helpful to understand the utmost benefit profile of patients from trabectedin. However, a limitation of our approach is that patients with GMI between 1 and 1.33 can also obtain substantial benefit from trabectedin, and have not been included in our analysis. Apart from the limited number of patients, the retrospective nature of this study makes impossible the analysis of other important issues such as toxicity.

It could also be worthwhile to validate the prognostic role of emerging blood cell rates as platelet/lymphoid or neuthophil/lymphoid cells in the context of advanced STS. Likewise GEISTRA, these rates can be routinely used in a daily basis and could complement our score [29].

Few studies explored molecular and genetic biomarkers as potential predictors of trabectedin efficacy [33]. In this sense, p53 and FAS expression predicted efficacy of trabectedin and doxorubicin at first line of ASTS [34]. Other studies investigated the nucleotide excision repair and homologous recombination DNA repair pathways, and found a significant correlation between better response and low BRCA1 mRNA expression and high ERCC1 or ERCC5 expression [35,36].

## 5. Conclusions

In conclusion, the GEISTRA score represents an easily applicable clinical tool that can be useful and reliable to better predict which patients with ASTS are the best candidates for the treatment with trabectedin in clinical practice.

## Figures and Tables

**Table 1:** Patients’ characteristics.

Patients Characteristics	Whole Series (*n* = 357) *n* (%)		Training Cohort *n* = 191	Validation Cohort *n* = 166	*p*
Age (Years)	Median (range): 50 (14–79)		50 (14–78)	51 (14–79)	0.094
Sex	Men	169 (47.3)	98 (51.3)	70 (42.2)	0.11
	Women	188 (52.7)	93 (48.7)	95 (57.6)	
Histology	L-sarcoma	193 (54.1)	96 (50.3)	97 (58.4)	0.14
	Leiomyosarcoma	113 (31.7))	57 (29.8)	56 (33.7)	0.49
	Liposarcoma	80 (22.4)	39 (20.4)	41 (24.7)	0.37
	Non-L-sarcoma	164 (45.9)	95 (49.7)	69 (41.6)	0.14
	UPS	37 (10.4)	27 (14.1)	10 (6)	0.014
	Other	127 (35.6)	68 (35.6)	59 (35.5)	1
	TR-sarcoma	92 (25.7)	50 (26.2)	42 (25.3)	0.904
	Non-TR-sarcoma	265 (74.2)	141 (73.8)	124 (74.7)	
FNCLCC tumor grade ^a^	1	44 (12.3)	23 (12)	21 (12.7)	0.47
	2	89 (24.9)	43 (22.5)	46 (27.7)	
	3	187 (52.4)	105 (55)	82 (49.4)	
	Missing	37 (10.4)	20 (10.5)	17 (10.2)	
Prior chemotherapy lines for advanced disease	0–1	154 (43.1)	75 (39.3)	79 (47.6)	0.24
	2	135 (37.8)	79 (41.3)	56 (33.7)	
	≥3	68 (19.0)	37 (19.4)	31 (18.7)	
Prior anthracycline administration setting	Adjuvant treatment	133 (37.3)	60 (31.4)	73 (44)	0.016
	First-line for advanced disease	224 (62.7)	131 (68.6)	93 (56)	
Stage at initial diagnosis	Nonmetastatic	276 (77.3)	146 (76.4)	130 (78.3)	0.52
	Metastatic disease	78 (21.8)	45 (23.6)	33 (19.9)	
	Missing	3 (0.8)	0 (0)	3 (1.8)	
Metastasis-free interval (months)	Median (range)	10.4 (0–177.2)	10.1 (0–174.5)	10.4 (0–177.2)	0.32
Karnofsky performance status	0–80	199 (55.7)	142 (55.3)	57 (58.8)	0.39
	>80	153 (42.8)	115 (44.7)	38 (39.2)	
	Missing	5 (1.4)	3 (1.2)	2 (2.1)	

^a^ Tumor specimens were classified according to the French Federation of Cancer Centers Sarcoma Group (FNCLCC) criteria. L-sarcoma, leiomyosarcoma and liposarcoma; TR-sarcoma, translocation-related sarcoma; UPS, undifferentiated pleomorphic sarcoma.

**Table 2:** Univariate and multivariate analysis of prognostic factors for worse TTP and OS.

	Univariate Analysis HR (95% CI)				Multivariate Analysis HR (95% CI)			
Prognostic Factor	Median TTP	*p*-Value	Median OS	*p*-Value	TTP	*p*-Value	OS	*p*-Value
Metastasis-free interval (months) - 0–10 - >10		0.001		0.002		<0.001		0.01
	2.8 (2.3–3.2)		8 (5.7–10.2)		1.6 (1.2–2.0)		1.4 (1.1–1.9)	
	4.1 (2.8–5.4)		14.7 (12–17.3)					
Metastatic at diagnosis - Yes - No		0.047		0.022		0.67		0.51
	3.5 (2.9–4)		11.7 (9.2–14.1)		0.9 (0.6–1.3)		1.1 (0.8–1.7)	
	4.4 (1.9–6.9)		18 (9–27)					
Age (years) - 0–50 - >50		0.16		0.081				
	3.7 (2.7–4.6)		13.5 (10.1–16.9)					
	3.5 (2.8–4.1)		11.3 (8.5–14.1)					
Sex - Male - Female		0.5		0.098				
	3.7 (3.1–4.4)		10.2 (7.7–12.7)					
	3.5 (2.6–4.5)		13.9 (11.3–16.4)					
Karnofsky PS - >80 - 0–80		<0.001		<0.001		<0.001		<0.001
	5.9 (4–7.7)		19.5 (16.8–22.2)					
	2.7 (2.3–3)		7.4 (5.4–9.4)		1.6 (1.2–2.1)		1.9 (1.5–2.5)	
FNCLCC tumor grade ^a^ - 1 - 2 - 3		<0.001		<0.001		0.015		0.027
	7.6 (5.4–9.8)		25.7 (13.8–37.6)					
	3.7 (2.2–5.2)		15.2 (10.9–19.4)					
	3 (2.5–3.5)		8.9 (6.1–11.6)		1.4 (1.1–1.8)		1.4 (1.0–1.8)	
Histology - L-sarcoma - Other		<0.001		<0.001		<0.001		<0.001
	5.1 (3.8–6.4)		17.9 (14.3–21.5)					
	2.8 (2.3–3.3)		7.3 (5.6–9)		1.9 (1.5–2.4)		1.8 (1.4–2.3)	
- L-sarcoma - UPS - Other	5.1 (3.8–6.4)	<0.001	17.9 (14.3–21.5)	<0.001				
	2.2 (1.6–3)		3.7 (2.5–4.9)					
	3.1 (2.6–3.6)		8 (6.2–9.8)					
- TR-sarcoma - Other	4.0 (3.0–3.9)	0.1	13.9 (8.4–19.3)	0.293				
	3.3 (2.7–3.8)		11.6 (9.3–13.8)					
Previous response - CR/PR - SD - PD		0.33		0.087				
	3 (2.4–3.5)		10.6 (8–13.2)					
	4.6 (3–6.1)		17 (12–22)					
	3.4 (3–4)		9.9 (5.9–13.8)					
Previous anthracycline ^b^ - Yes - No		0.15		1				
	3.8 (2.7–4.8)		13.1 (8.9–17.3)					
	3.4 (2.8–3.9)		11.8 (9.3–14.2)					
Previous gemcitabine ^b^ - Yes - No		0.091		0.83				
	3.4 (2.9–3.9)		12.2 (9.3–15.2)					
	3.8 (2.9–4.7)		12 (9.4–14.6)					
Previous chemotherapy line for advanced disease - 1 - 2 - ≥ 3		0.12		0.48				
	3.8 (2.8–4.8)		12.2 (8.8–15.7)					
	3.8 (3.1–4.5)		13.1 (10.8–15.5)					
	3.3 (2.5–4)		9.2 (5.4–13.1)					

^a^ Tumor specimens were classified according to the French Federation of Cancer Centers Sarcoma Group (FNCLCC) criteria. ^b^ Drugs given immediately prior to treatment with trabectedin. CI, confidence interval; CR, complete response; OS, overall survival; HR, hazard ratio; PD, progressive disease; PR, partial response; PS, performance status; SD, stable disease; TR-sarcoma, translocation-related sarcoma; TTP, time to progression.

**Table 3:** Relation between the GMI and other activity end-points in the training cohort.

Outcome Endpoints	GMI 0–1.33	GMI > 1.33	*p*-Value
Response to trabectedin - Complete/partial response - Stable disease - Progressive disease			<0.001
	7 (6.4%)	14 (20.5%)	
	21 (19.1%)	39 (57.4%)	
	82 (74.5%)	15 (22.1%)	
Median OS from trabectedin (95% CI)	6.4 (4–8.9)	25.2 (14–36.4)	<0.001
Median OS from initial diagnosis (95% CI)	34.8 (30.2–39.4)	64.6 (51.1–78.1)	<0.001
Median OS from metastatic disease (95% CI)	23 (17.3–28.6)	32.7 (27.8–37.7)	<0.001
Median TTP trabectedin (95% CI)	2.3 (2–2.6)	8.2 (6.2–10.1)	<0.001

GMI, growth modulation index; OS, overall survival; TTP, time to progression.

**Table 4:** Univariate and multivariate analysis of variables in relation to growth modulation index (GMI) in the training cohort.

Prognostic Factors	Univariate Analysis			Multivariate Analysis	
	GMI 0–1.33	GMI > 1.33	*p*-Value	Odds Ratio (95% CI)	*p*-Value
Metastasis-free interval (months) - 0–5 - >5			0.003		0.01
	52 (46%)	13 (22%)			
	62 (54%)	46 (78%)		2.81 (1.29–6.16)	
Metastatic at diagnosis - Yes - No			0.013		
	36 (29%)	9 (13%)			
	87 (71%)	59 (87%)			
Age (years) - 0–50 - >50			1		
	64 (52%)	35 (51%)			
	59 (48%)	33 (48%)			
Sex - Male - Female			0.37		
	60 (49%)	38 (56%)			
	63 (51%)	30 (44%)			
Karnofsky PS - 0–80 - >80			0.039		0.044
	73 (61%)	29 (43%)			
	47 (39%)	39 (57%)		2.14 (1.02–4.5)	
FNCLCC tumor grade ^a^ - 1 - 2 - 3			0.55		
	13 (12%)	10 (16%)			
	30 (27%)	13 (21%)			
	67 (61%)	38 (62%)			
Histology - L-sarcoma - Other			0.023		0.031
	54 (44%)	42 (62%)		2.26 (1.08–4.74)	
	69 (56%)	26 (38%)			
Histology - L-sarcoma - UPS - Other			0.032		
	54 (44%)	42 (62%)			
	22 (18%)	5 (7%)			
	47 (38%)	21 (31%)			
Histology - TR-sarcoma - Other			0.458		
	61(25%)	31(26.3%)			
	178(75%)	87(73.7%)			
Previous best response - CR/PR - SD - PD			<0.001		
	32 (26%)	5 (8%)			
	34 (28%)	11 (17%)			
	55 (45%)	49 (75%)			
Previous best response - CR/PR/SD - PD			<0.001		<0.001
	66 (54%)	16 (25%)			
	55 (45%)	49 (75%)		4.96 (2.24–10.97)	
Previous anthracycline - Yes - No			0.26		
	35 (28%)	25 (37%)			
	88 (71%)	43 (63%)			
Gemcitabine - Yes - No			0.54		
	54 (44%)	26 (38%)			
	69 (56%)	42 (62%)			
Number of prior lines for advanced disease - 1 - 2 - ≥3			0.022		
	41 (33%)	34 (50%)			
	52 (42%)	27 (40%)			
	30 (24%)	7 (10%)			

^a^ Tumor specimens were classified according to the French Federation of Cancer Centers Sarcoma Group (FNCLCC) criteria. CR, complete response; GMI, growth modulation index; PD, progressive disease; PR, partial response; PS, performance status; SD, stable disease; TR-sarcoma, translocation-related sarcoma; UPS, undifferentiated pleomorphic sarcoma.

**Table 5:** Correlation between GEISTRA score and time-to-event outcomes from trabectedin therapy in the training and validation cohort.

		Training Cohort				Validation Cohort			
		TTP		OS		TTP		OS	
GEISTRA Staging Group	GEISTRA Score	mTTP (95% CI)	*p*-Value	mOS (95% CI)	*p*-Value	mTTP (95% CI)	*p*-Value	mOS (95% CI)	*p*-Value
0	0–1	6.4 (3.8–8.9)	<0.001	19 (16.4–21.7)	<0.001	6.5 (1.7–11.3)	0.017	24.9 (18.4–31.3)	0.004
1	2–4	2.5 (2–3)		7.4 (5.7–9.2)		3.1 (2.3–3.9)		10.5 (5.3–15.7)	
Whole series		3.4 (2.8–4)		11.2 (8.9–13.5)		3.4 (2.8–4)		11.2 (8.9–13.5)	

CI, confidence Interval; TTP: Time to Progression; OS: Overall Survival; mTTP: Median TTP; mOS: median OS.

## Data Availability

The datasets used and/or analyzed during the current study are available from the corresponding author on reasonable request.

## Supplementary Materials

The following are available online at https://www.mdpi.com/2072-6694/13/4/792/s1, Table S1: Comparative table with outcome measures of different studies of ASTS treated with trabectedin.
