# Supplementary material for: A Growth Modulation Index-Based GEISTRA Score as a New Prognostic Tool for Trabectedin Efficacy in Patients with Advanced Soft Tissue Sarcomas: A Spanish Group for Sarcoma Research (GEIS) Retrospective Study
Source: Cancers (Basel). 2021 Feb 14;13(4):792. doi: 10.3390/cancers13040792 (PMC7917652; doi:10.3390/cancers13040792)
Supplement: Supplementary file 1 [file cancers-13-00792-s001.pdf]

Supplementary Materials

A Growth Modulation Index-Based GEISTRA Score as a New Prognostic Tool for Trabectedin Efficacy in Patients with Advanced Soft Tissue Sarcomas: A Spanish Group for Sarcoma Research (GEIS) Retrospective Study

Javier Martínez-Trufero, Luis Miguel De Sande-González, Pablo Luna, Javier Martin-Broto, Rosa Álvarez, Gloria Marquina, Roberto Diaz-Beveridge, Andrés Poveda, Juana María Cano [9], Josefina Cruz-Jurado, Antonio López Pousa, María Angeles Vaz Salgado Claudia M. Valverde-Morales, Isabel Sevilla, Jerónimo Martínez-García, Jordi Rubio-Casadevall, Ana De Juan, Juan Antonio Carrasco, David da Silva Moura Ibon Gurruchaga-Sotes and Antonio Gutiérrez

Table S1. Comparative table with outcome measures of different studies of ASTS treated with trabectedin.

Studies	Study Type	Sarcoma Subtypes	*n*	Median TTP/PFS (Months)	Median OS (Months)	ORR (%)	SD (%)	DCR (%)
Buonadonna et al. [1]	Phase IV trial	All STS	218	5.9 (4.9–7.8) *	21.3 (18.8–24.3	26.6 (20.9–33)	39	65.6 (58.9–71.9)
Demetri et al. [2]	Phase II trial	L-sarcoma	136	3.7 (2.1-5.4)	13.9 (12.5–18.6)	5.6 (2.3–11.2)	52.8	58.4 (49.3–67.2)
Demetri et al. [3]	Phase III trial	L-sarcoma	345	4.2*	13.7 (12.2–16)	9.9 (6.9–13.5)	51	61.2
Kawai et al. [4]	Phase II trial	TR-sarcoma	37	5.6 (4.1–7.5) *	NR (12.8–NR)	8	57	65
Samuels et al. [5]	Retrospective	All STS	807	-	11.9	5.9 (4.4–7.8)	43	48.5
		L-sarcoma	476	-	16.2 (14.1–19.5)	6.9 (4.8–9.6)	47	54.2
		Non L-sarcoma	331	-	8.4 (7.1–10.7)	4.0 (2.1–6.8)	33.8	37.7
Le Cesne et al. [6]	Retrospective	All STS	804	4.4 (3.9–4.9) *	12.2 (11.0–13.3)	16.5	50.1	66.7
		L-sarcoma	482	5.7 (4.9–6.5) *	15.0 (13.2–16.8)	18.6	54	72.6
De Nonneville et al. [7]	Retrospective	All STS	87	4.17 (3.1–5.2) *	26.7(14.3–39.1)			
Kobayashi el al. [8]	Retrospective	All STS	140	3.7 (2.8-5.7) *	16.4(11.5-21.2)	7.9	41.9	49.8
Present study	Retrospective	All STS	357	3.5 (2.8-4.0)	12.0 (10-13.9)	11.5	35.4	48.0
		L-sarcoma	193	5.1 (3.8-6.4)	17.9 (14.3-21.5)	15.1	37.3	52.4
		TR-sarcoma	92	4.0 (3.0-3.9)	13.9 (8.4-19.3)	11.5	30.4	41.9

* Data correspond to median PFS. DCR, disease control rate; L-sarcoma, liposarcoma and/or leiomyosarcoma; NR, not reached; ORR, objective response rate; OS, overall survival; SD, stable disease; STS, soft tissue sarcoma; TTP, time to progression; TR-sarcoma, translocation-related sarcoma.

References

1 Buonadonna, A.; Benson, C.; Casanova, J.; Kasper, B.; Lopez Pousa, A.; Mazzeo, F.; Brodowicz, T.; Penel, N. A noninterventional, multicenter, prospective phase IV study of trabectedin in patients with advanced soft tissue sarcoma. *Anti-Cancer Drugs* **2017**, *28*, 1157–1165, doi:10.1097/CAD.0000000000000560.

2. Demetri, G.D.; Chawla, S.P.; von Mehren, M.; Ritch, P.; Baker, L.H.; Blay, J.Y.; Hande, K.R.; Keohan, M.L.; Samuels, B.L.; Schuetze, S.; et al. Efficacy and safety of trabectedin in patients with advanced or metastatic liposarcoma or leiomyosarcoma after failure of prior anthracyclines and ifosfamide: Results of a randomized phase II study of two different schedules. *J. Clin. Oncol.* **2009**, *27*, 4188–4196, doi:10.1200/jco.2008.21.0088.

3. Demetri, G.D.; von Mehren, M.; Jones, R.L.; Hensley, M.L.; Schuetze, S.M.; Staddon, A.; Milhem, M.; Elias, A.; Ganjoo, K.; Tawbi, H.; et al. Efficacy and Safety of Trabectedin or Dacarbazine for Metastatic Liposarcoma or Leiomyosarcoma After Failure of Conventional Chemotherapy: Results of a Phase III Randomized Multicenter Clinical Trial. *J. Clin. Oncol.* **2016**, *34*, 786–793, doi:10.1200/JCO.2015.62.4734.
4. Kawai, A.; Araki, N.; Sugiura, H.; Ueda, T.; Yonemoto, T.; Takahashi, M.; Morioka, H.; Hiraga, H.; Hiruma, T.; Kunisada, T.; et al. Trabectedin monotherapy after standard chemotherapy versus best supportive care in patients with advanced, translocation-related sarcoma: A randomised, open-label, phase 2 study. *The Lancet. Oncology* **2015**, *16*, 406–416, doi:10.1016/S1470-2045(15)70098-7.
5. Samuels, B.L.; Chawla, S.; Patel, S.; von Mehren, M.; Hamm, J.; Kaiser, P.E.; Schuetze, S.; Li, J.; Aymes, A.; Demetri, G.D. Clinical outcomes and safety with trabectedin therapy in patients with advanced soft tissue sarcomas following failure of prior chemotherapy: Results of a worldwide expanded access program study. *Ann. Oncol.* **2013**, *24*, 1703–1709, doi:10.1093/annonc/mds659.
6. Le Cesne, A.; Ray-Coquard, I.; Duffaud, F.; Chevreau, C.; Penel, N.; Bui Nguyen, B.; Piperno-Neumann, S.; Delcambre, C.; Rios, M.; Chaigneau, L.; et al. Trabectedin in patients with advanced soft tissue sarcoma: A retrospective national analysis of the French Sarcoma Group. *Eur. J. Cancer* **2015**, *51*, 742–750, doi:10.1016/j.ejca.2015.01.006.
7. De Nonneville A, Barbolosi D, Andriantsoa M, El-Cheikh R, Duffaud F, Bertucci F, Salas S. Validation of Neutrophil Count as An Algorithm-Based Predictive Factor of Progression-Free Survival in Patients with Metastatic Soft Tissue Sarcomas Treated with Trabectedin. *Cancers* **2019**, *11*, 432, doi:10.3390/cancers11030432.
8. Kobayashi H, Iwata S, Wakamatsu T, Hayakawa K, Yonemoto T, Wasa J, Oka H, Ueda T, Tanaka S. Efficacy and safety of trabectedin for patients with unresectable and relapsed soft-tissue sarcoma in Japan: A Japanese Musculoskeletal Oncology Group study. *Cancer* **2020**, *126*, 1253–1263, doi:10.1002/cncr.32661.
